# Dysregulation of miR-638 in diabetic nephropathy and its role in inflammatory response

**DOI:** 10.1186/s13098-021-00744-2

**Published:** 2021-10-29

**Authors:** Mei Lin, Dan Song, Suo Zhang, Ping Li

**Affiliations:** Department of Nephrology, University of Chinese Academy of Sciences Shenzhen Hospital, 4221 Songbai Road, Shenzhen, 518000 Guangdong China

**Keywords:** miR-638, Differential diagnosis, Diabetic nephropathy

## Abstract

**Background:**

MicroRNA (miRNA) can be used as a biomarker for the early diagnosis of diabetic nephropathy (DN). The purpose of this study was to evaluate the diagnostic value of miR-638 in DN and to analyse its regulatory effect on inflammation.

**Methods:**

This retrospective study involved 98 subjects, including non-diabetic healthy controls (n = 30), patients with type 2 diabetes (T2DM, n = 36) without complications and patients with DN (n = 32). After the anthropometric and biochemical evaluation, serum miR-638 levels were assessed by real-time reverse transcription-polymerase chain reaction (qRT-PCR). The levels of inflammatory cytokines (interleukin [IL]-1β, IL-6, and tumor necrosis factor-alpha [TNF-α]) were detected using enzyme-linked immunosorbent assay. The Spearman correlations were used to analyze the correlation between miR-638 and urinary albumin excretion (UAE), estimated glomerular filtration rate (eGFR), and inflammatory factors. Furthermore, the receiver operating characteristic (ROC) curve was used to measure the diagnostic value of miR-638 in DN. Human mesangial cells (HMCs) were treated with normal glucose (NG, 5.5 mM glucose), high glucose (HG, 30 mM glucose), or high osmotic pressure solution (HO, 5.5 mM glucose + 24.5 mM mannitol) in vitro to simulate the hyperglycamic state in vivo. Subsequently, the HMCs were transfected with miR-638 mimics to regulate the level of miR-638 in the cells and detect its regulation on cell inflammation and proliferation.

**Results:**

Compared with healthy controls and patients with T2DM, serum miR-638 in patients with DN was significantly lower. The reduced miR-638 expression has a significant diagnostic value, which can significantly distinguish patients with DN from healthy controls or patients with T2DM. Inflammatory factors were significantly upregulated in patients with DN and negatively correlated with miR-638 levels. In addition, miR-638 was negatively correlated with UAE and positively correlated with eGFR. HG decreased the level of miR-638 and promoted the expression of inflammatory factors and proliferation in HMCs. However, miR-638 mimic significantly decreased the levels of inflammatory factors and inhibited the proliferative ability induced by HG.

**Conclusions:**

Serum miR-638 expression was low in DN and can be a potentially valuable biomarker for DN. This miRNA seems to influence inflammatory responses and participate in the progression of DN by regulating proliferation.

## Introduction

The incidence of type 2 diabetes mellitus (T2DM) is rapidly increasing worldwide and is estimated to increase to 10.4% by 2040 [1]. Diabetic nephropathy (DN) is one of the most important chronic complications of T2DM, and the main cause of end-stage renal disease (ESRD) and death in patients with diabetes [2]. More than 40% of patients with diabetes eventually develop DN [3], with the prevalence of DN in China being 20%–30% [4]. Furthermore, DN is a powerful risk factor for complications such as cardiovascular disease (myocardial infarction, stroke, atherosclerosis, and hypertension), neuropathy, and kidney disease [5]. This disease is believed to be caused by factors such as genetic susceptibility, glucose metabolism disorder, renal hemodynamics changes, oxidative stress, and cytokines [6]. In addition, the occurrence of inflammation is also an important pathophysiological mechanism of DN [7, 8]. Currently, the routine diagnosis of DN is based on urinary albumin excretion (UAE) and estimated glomerular filtration rate (eGFR) [9]. However, there is a possibility of these abnormalities occurring in different forms of kidney injury [10]. Therefore, there is an urgent need for new, highly specific, rapid, and non-invasive biomarkers for accurate diagnosis and treatment of DN.

MicroRNAs (miRNAs) are non-coding RNA molecules composed of 18–22 nucleotides, which are involved in the post-transcriptional regulation of genes. Previous studies have reported that miRNAs, as powerful regulators of various diseases, may play a key role in the occurrence and development of diseases such as DN [11, 12]. Moreover, owing to their stability, repeatability and consistency, miRNAs have become potential biomarkers for the prediction and diagnosis of various diseases including DN [13]. miRNA-27b-3p and miR-1228-3p can be used as biomarkers for DN fibrosis progression [10]. miR-377 and miR-246a are early diagnostic markers of type 1 DM in pediatric patients and may be associated with atherosclerosis [14].

Denis et al. analysed the urinary exosomes of healthy controls, T2DM and DN in 2016 and observed 14 differentially expressed miRNAs, including miR-638 [15]. Furthermore, multiple studies have confirmed the abnormal expression of miR-638 in DN complications. For instance, miR-638 is abnormally expressed in the kidneys of patients with lupus nephritis and is associated with the severity of clinical disease [16]. The decrease in serum miR-638 levels is significantly related to the vulnerability of the atherosclerotic plaque and can be used as a biomarker for plaque vulnerability and ischemic stroke [17]. However, its role in DN has not been studied and reported.

In light of all this evidence, we aimed to clarify the potential clinical value of miR-638 in patients with DN and try to analyse the potential correlation between its expression level and inflammation.

## Materials and methods

### Subject sample

A total of 68 patients with T2DM admitted to the University of Chinese Academy of Sciences Shenzhen Hospital from June 2017 to December 2018 were selected and their diagnoses were based on the criteria of the American Diabetes Association (ADA) [18]. The patients with T2DM were divided into the T2DM group and the DN group. The inclusion criteria of both groups of patients are as follows: (1) patients age > 18 years, (2) patients who fulfilled the diagnostic criteria of the ADA of 2016 [18]; (3) patients who presented with a history of diabetes for more than a year and (4) patients without diabetes-related complications such as diabetic retinopathy and with a normal eGFR and UAE in the routine diagnosis of DN. The inclusion criteria of the DN group induced additional criteria such as (1) urinary protein/creatinine ratio > 30 mg/g, persistent urinary protein or renal function decline (GFR < 60 mL/min/1.73m^2^), and (2) biopsy confirmed that the renal disease is caused by diabetes. Patients with infectious diseases, chronic systemic inflammatory diseases, blood diseases, autoimmune diseases, malignant tumors and cardiovascular diseases were excluded. In addition, 30 age-matched healthy participants from a health examination center were selected as the control group. The inclusion criteria were as follows: (1) no history of diabetes, that is, patients who do not meet the ADA criteria for the diagnosis of diabetes and (2) normal blood glucose, renal function, and renal ultrasonography Urine and venous blood samples were collected from all subjects after a night of fast for clinical and laboratory tests. The statistics of the following parameters were measured: (1) anthropometric data including age, gender, body mass index (BMI), systolic blood pressure (SBP) and diastolic blood pressure (DBP) and (2) analytical data including lipid parameters (total cholesterol [TC] and triglyceride), fasting blood glucose (FBG), glycosylated haemoglobin (HbA1c) expression percentage, and renal parameters (UAE and eGFR) (Table 1).

**Table 1:** Comparison of the baseline data of study objects

Parameters		Health (n = 30)	T2DM (n = 36)	DN (n = 32)
Age (year)		54.31 ± 4.81	55.05 ± 6.66	55.05 ± 6.67
Gender (male/female)		14/16	20/16	19/13
BMI (kg/m^2^)		26.27 ± 2.53	27.44 ± 5.25	27.44 ± 5.25
SBP (mmHg)		128.73 ± 8.35	126.24 ± 18.02	126.24 ± 13.63
DBP (mmHg)		75.07 ± 11.07	78.04 ± 17.20	77.55 ± 10.58
TC (mg/dL)		171.50 ± 19.34	178.09 ± 13.86	177.27 ± 15.68
Triglyceride (mg/dL)		149.45 ± 19.53	167.10 ± 24.55	158.05 ± 14.37
FBG (mmol/L)		5.33 ± 1.64	8.04 ± 1.53	9.23 ± 1.90* ^#^
HbA1c (%)		5.54 ± 1.04	8.78 ± 2.01	7.59 ± 2.33 *
UAE (mg/24 h)		–	7.25 ± 2.34	77.16 ± 30.88* ^#^
eGFR (mL/min)		–	102.84 ± 10.35	67.82 ± 16.56 * ^#^
IL-1β (pg/mL)		–	7.06 ± 2.99	14.16 ± 3.77* ^#^
IL-6 (pg/mL)		–	7.54 ± 2.26	9.60 ± 4.38* ^#^
TNF-α (pg/mL)		–	8.52 ± 2.99	10.32 ± 2.34 * ^#^

BMI, body mass index; TC, total cholesterol; FBG fasting blood glucose; HbA1c, glycosylated hemoglobin; UAE, urinary albumin excretion; eGFR, estimated glomerular filtration rate; T2DM, Type 2 diabetes mellitus; DN, diabetic nephropathy

* *P* < 0.05, compared with Healthy control; ^##^ *P* < 0.05, compared with T2DM patients

This study is based on the Declaration of Helsinki, and the proposals have been approved by the ethics committee of the University of Chinese Academy of Sciences Shenzhen Hospital. All subjects signed informed consent forms.

### Cell culture and transfection

Human mesangial cells (HMCs) were purchased from the Chinese Type Culture Collection (CTCC) and cultured in DMEM containing 10% fetal bovine serum (FBS) and 1% penicillin/streptomycin. miR-638 mimic and negative control were transfected with Lipofectamine 3000 respectively, and the new medium was replaced 6 h later according to the manufacture’s requirements. miR-638 mimic leads to over-expression of the miRNA, while they mimic NC as a negative control neither increases nor decreases miRNA-638 levels. Synthetic miR-638 mimic and mimic NC were purchased from Guangzhou RiboBio Co. In vitro, cell glucose culture was carried out at different concentrations, including normal glucose (NG, 5.5 mM glucose), high osmotic pressure solution (HO, 5.5 mM glucose + 24.5 mM mannitol), and high glucose (HG, 30 mM glucose). The miRNA levels were measured under a time gradient.

### Real-time quantitative polymerase chain reaction (RT-qPCR) analysis

RT-qPCR was used to analyze the mRNA level of miR-638. Total RNA in serum and cell lines was extracted by QIAamp RNA blood Kit and miRNA Purification Kit. The quality and quantity of RNA were monitored by a spectrophotometer. Then, the RNA was reverse transcribed to cDNA using the miRcute Plus miRNA First-strand cDNA Kit according to the manufacturer’s instructions. Finally, the RT-qPCR reaction was performed using the miRcute Plus miRNA qPCR (SYBR Green) Kit and on the ABI StepOne Plus real-time PCR instrument. U6 was used as the internal reference gene, and the relative expression level of miR-638 was calculated by the method of 2^−ΔΔCt^.

### Enzyme-linked immunoreactivity assay (ELISA)

The levels of tumor necrosis factor α (TNF-α), interleukin-1β (IL-1β), and interleukin-6 (IL-6) in subjects' serum and cells were determined by ELISA Kit. The cell supernatant was cultured 48 h after transfection and the absorbance at 450 nm was measured according to the manufacturer's instructions. Human ELISA kits (TNF-α, ab181421; IL-1β, ab108865; IL-6, ab46027) were all purchased from Abcam lnc.

### Cell proliferation assay

Cell Counting Kit-8 (CCK-8) was used to detect the HMCs proliferation. After transfection with miR-638 mimic, the cells were seeded into 96-well plates. Add medium containing different concentrations of glucose to continue culturing cells. What’s more, CCK-8 reagents were added at 0, 24, 48, 72 h, and followed by incubation at 37 °C for 1 h. Finally, the change of absorption value at 450 nm was detected.

### Statistical analysis

SPSS 23.0 and GraphPad 6.0 software were used for statistical analysis of the data. And they were recorded as the mean ± SD of at least three dependent experiments. According to the normal distribution of data, the parametric student t-test or the non-parametric Mann–Whitney's U-test is performed on the data. Kruskal Wallis test and Dunn’s multiple comparison post hoc test were used for data of more than two groups. Correlation between miR-638 level and inflammatory factors was analyzed by the Spearman correlation analysis. The receiver operating characteristic (ROC) curve evaluated the diagnostic value of miR-638 for diseases and calculated the area under the curve (AUC). *P* < 0.05 was considered statistically significant.

## Results

### Anthropometric and biochemical parameters

The clinical data of the subjects were evaluated, and the results are presented in Table 1. There were no significant differences in age, gender, BMI, SBP, DBP, TC levels and triglyceride levels among the three groups (*P* > 0.05). However, FBG, IL-1β, IL-6, and TNF-α levels and UAE were significantly increased, whereas eGFR was significantly decreased compared with patients with T2DM (*P* < 0.05).

### Serum miR-638 was downregulated in patients with DN patients

**Fig. 1:**
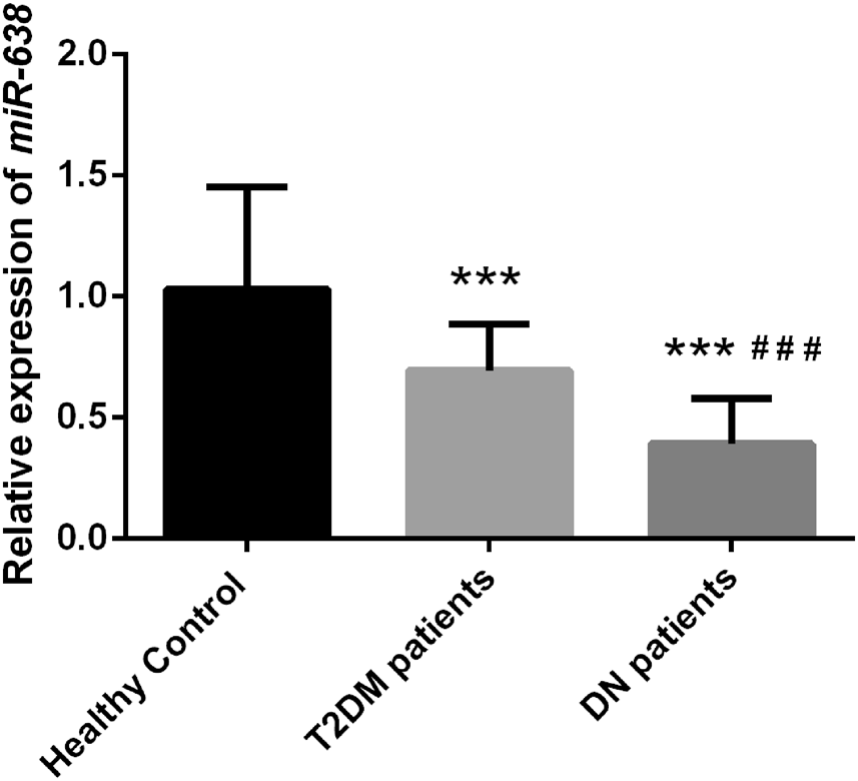
The expression level of serum micro-ribonucleic acid (miRNA) miR-638 in different patients. The level of serum miR-638 in patients with diabetic nephropathy (DN) is significantly lower than that of patients with type 2 diabetes mellitus (T2DM) and healthy controls. *** *P* < 0.001, compared with Healthy control; ^# # #^ *P* < 0.001, compared with patients with type 2 diabetes mellitus (T2DM) patients

Subsequently, serum miR-638 levels were measured. When compared with healthy control, serum miR-638 levels were significantly reduced in both patients with T2DM and DN (*P* < 0.05, Fig. 1). Compared with the patients in the other two groups, patients with DN had the lowest serum miR-638 levels (*P* < 0.05, Fig. 1). The results suggested that the dysregulation of miR-638 may be involved in the progression of DN.

### Serum miR-638 was correlated with clinical indicators of patients with DN

To verify our hypothesis, we further analysed the correlation between serum miR-638 levels and UAE, eGFR, and inflammatory factors. As presented in Table 2, serum miR-638 was significantly negatively correlated with UAE (r = − 0.6886, *P* < 0.05), IL-1β (r = − 0.6291, *P* < 0.05), and moderately correlated with IL-6 (r = − 0.5214, *P* < 0.05) and TNF-α (r = − 0.5025, *P* < 0.05). However, serum miR-638 was significantly positivity correlated with eGFR (r = 0.6448, *P* < 0.05). The results suggested that serum miR-638 was closely associated with the commonly used DN diagnostic markers and pro-inflammatory factors.

**Table 2:** Correlation of miR-638 relative expression with clinical characteristics

Parameters	MiR-638 expression	
	*P* value	Correlation coefficient (*r*)
UAE	0.000	− 0.6886
eGFR	0.000	0.6448
IL-1β	0.000	− 0.6291
IL-6	0.0022	− 0.5214
TNF-α	0.0030	− 0.5028

### Serum miR-638 has a high diagnostic value in patients with DN

**Fig. 2:**
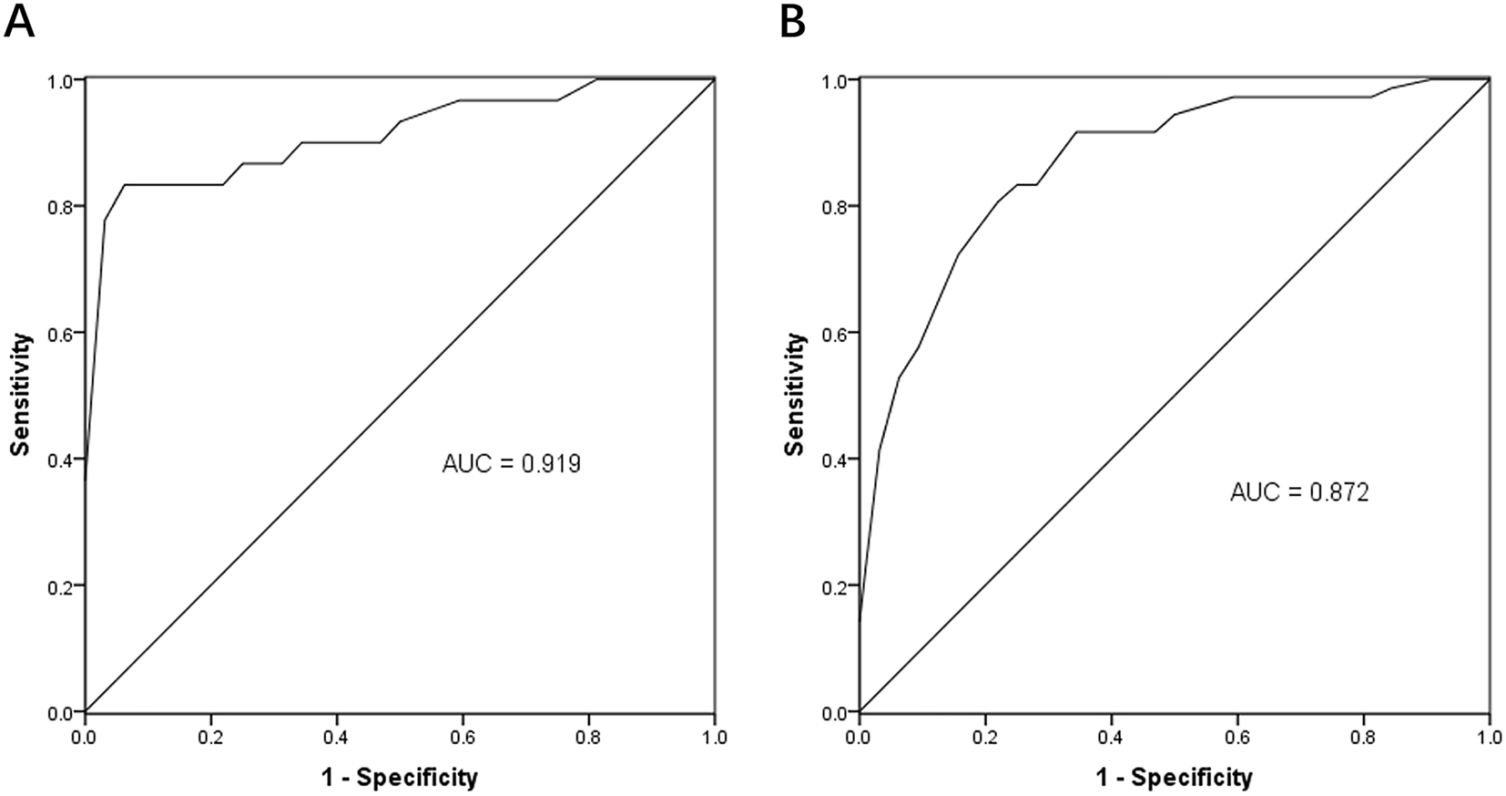
Receiver operating characteristic (ROC) is was used to evaluate the diagnostic value of serum miR-638 in different patients. **A** Patients with diabetic nephropathy (DN) from a healthy control. **B** Patients with diabetic nephropathy (DN) patients from patients with type 2 diabetes mellitus (T2DM) patients

Receiver operating characteristic (ROC) analysis was performed based on the serum miR-638 levels of subjects to evaluate the diagnostic value of miR-638 in patients with DN. The results revealed that serum miR-638 differentiated between patients with DN patients and healthy controls (Fig. 2A). The area under the curve (AUC) was 0.919, the cut-off value was 0.715 and the sensitivity and specificity were 83.3% and 96.87%, respectively. Moreover, it was observed from the ROC curve analysis also found that serum miR-638 significantly distinguished patients with DN from patients with T2DM (Fig. 2B). The AUC cut-off value, sensitivity, and specificity were 0.872, 0.57, 80.06% and 84.37%, respectively. The ROC curve analysis suggested that serum miR-638 can be a valuable biomarker for diagnosing patients with DN.

### Upregulation of miR-638 reduced the levels of HG-induced pro-inflammatory factors and the ability of proliferation

**Fig. 3:**
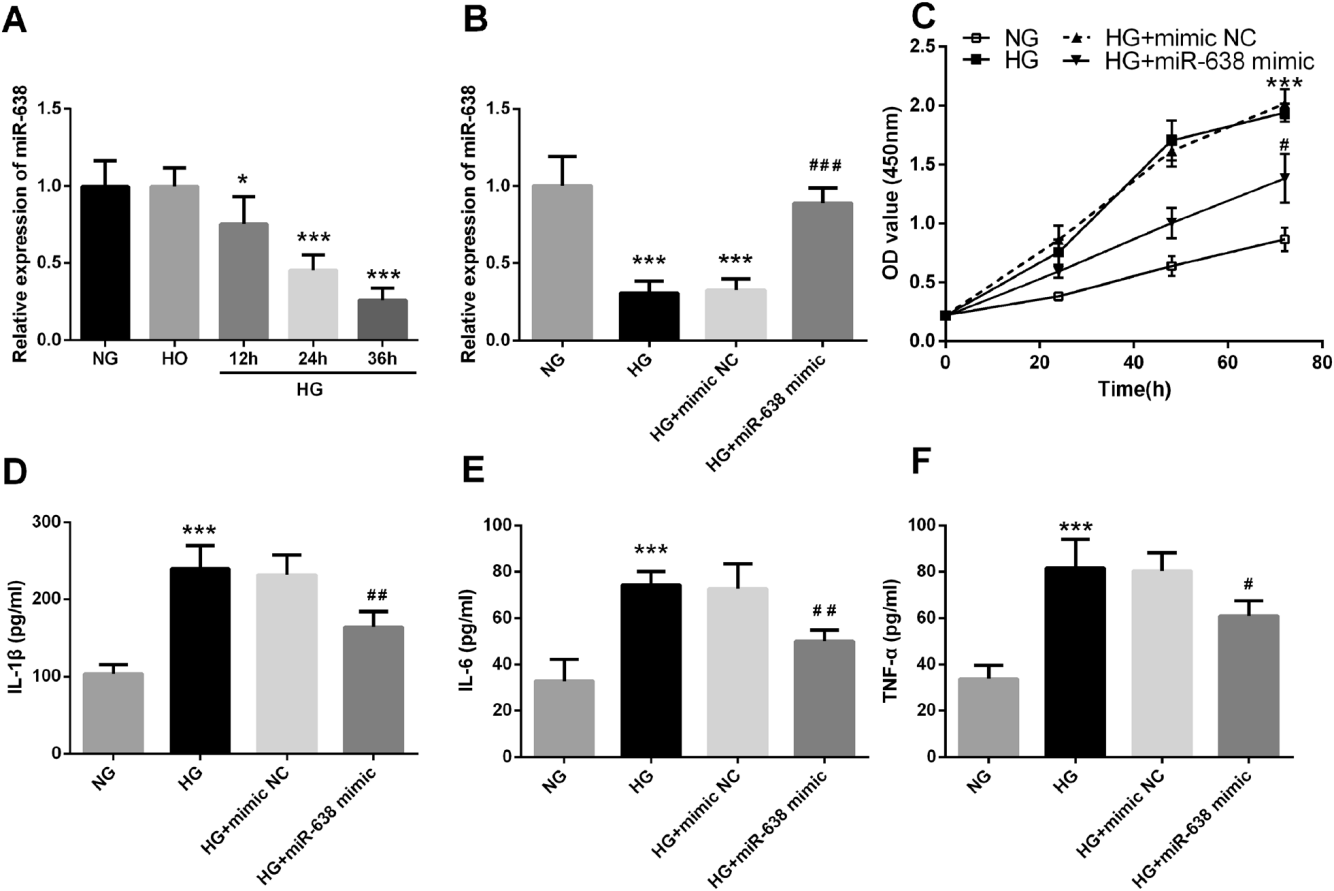
Effects of microRNA-638 (miR-638) upregulation on high glucose-induced pro-inflammatory factors and proliferation. **A** Real-time reverses transcription-polymerase chain reaction (qRT-PCR) is used to detect the expression level of miR-638 in high glucose-induced human mesangial cells (HMCs). **B** The expression level of miR-638 after transfection with miR-638 mimic and high glucose (HG) stimulation of human mesangial cells (HMCs). **C** Cell counting kit-8 (CCK-8) is used to detect the proliferation of HMCs transfected with miR-638 mimic and cultured with high glucose (HG)**. D–F** Enzyme-linked immunosorbent assay (ELISA) is used to detect the level of inflammatory cytokines. * *P* < 0.05, *** *P* < 0.001, compared with normal glucose (NG); ^# # #^ *P* < 0.05, ^# #^ *P* < 0.01, ^# # #^ *P* < 0.05, compared with high glucose (HG)

To further investigated the potential effect of miR-638 on DN, human mesangial cells (HMCs) were cultured in high glucose (HG) for 12, 24, and 36 h. Real-time reverse transcription-polymerase chain reaction (qRT-PCR) revealed that miR-638 levels decreased in a time-dependent manner after HG treatment as compared with normal glucose (NG) treatment (*P* < 0.05, Fig. 3A). After transfecting the cells with miR-638 mimics, the level of miR-638 was regulated in vitro and was significantly increased (*P* < 0.05, Fig. 3B). It was further observed that HG significantly increased pro-inflammatory cytokines levels and the proliferation ability in HMCs. This promoting effect was significantly eliminated by increasing miR-638 (*P* < 0.05, Fig. 3C–F).

## Discussion

Our results determined that miR-638 was downregulated in DN. Moreover, it was confirmed for the first time that low miR-638 expression can distinguish patients with DN from patients with T2DM and healthy controls, therefore, miR-638 and can be used as a diagnostic biomarker for DN. In addition, miR-638 may participate in DN by regulating proliferation and inflammation, thus providing a new therapeutic target for the treatment of DN.

DN is the most common complication in patients with diabetes patients, often leading to ERSD. Approximately 40% of patients with diabetes patients eventually develop DN and kidney disease [19]. The main pathological features of DN are glomerular hypertrophy, hyperplasia, thickened basement membrane and increased extracellular matrix. DN gradually develops into glomerular sclerosis, interstitial fibrosis and loss of kidney function, eventually leading to chronic renal failure, which remarkably affects the quality of life and endangers the life of patients [20]. Currently, the early diagnosis and detection indicators commonly used in clinical practice cannot accurately reflect the condition of DN. Although renal biopsy can diagnose and determine disease progression, it is invasive and increases the risk of infection [21]. Therefore, there is an urgent need to discover new, highly specific, rapid, and non-invasive biomarkers to accurately identify patients with DN, and lay the foundation for their treatment.

As well-known biomarkers, miRNAs have been widely used in the clinical diagnosis and treatment of DN [22]. The specific changes of miRNA in kidney tissue, serum and urine can regulate inflammation, metabolic abnormalities, immune response and fibrosis through different signaling pathways and targets, thereby affecting the occurrence and development of DN [23]. Serum miR-21 is closely related to the structure and function of the kidney and is a potential biomarker for the diagnosis of DN [24]. MiR-451 can be used as an early predictor of chronic diseases in DN [25].

Previous studies have demonstrated that miR-638 is abnormally expressed in DN. Such as, Conserva et al. evaluated the differential expression of nine miRNAs in DN, T2DM, and normal kidney biopsy tissues, including miR-638 [10]. In 2016, Denis et al. analysed urinary exosomes in healthy controls, T2DM and DN, and observed 14 abnormally expressed miRNAs, including miR-638 [15]. In addition, miR-638 is also has dysregulated in cardiovascular disease, a common complication of DN. For instance, low serum miR-638 levels are significantly associated with plaque vulnerability in patients with carotid artery stenosis [17]. Overexpression of miR-638 can significantly reduce the cardiomyocyte damage induced by hypoxia and reoxygenation [26]. In addition, miR-638 can significantly inhibit the calcification of human aortic valve interstitial cells [27]. Therefore, we speculated that miR-638 played an important role in DN. To verify our hypothesis, we first detected the serum miR-638 expression levels of subjects. The results revealed that miR-638 was significantly decreased in patients with DN and T2DM, which was consistent with the results of studies performed by Conserva et al. and Denis et al. [10, 15]. In addition, we evaluated the diagnostic value of miR-638. The results revealed that miR-638 significantly distinguished patients with DN from those with T2DM or healthy controls reveal a high diagnostic value.

To further study the effect of miR-638 on DN, we analyzed the relationship between miR-638 and UAE and eGFR. The results revealed that miR-638 was negatively correlated with UAE, and positively correlated with eGFR. Previous studies have demonstrated that UAE is a marker of early DN and endothelial damage [28]. Simultaneously, UAE is an important marker of renal insufficiency and an important predictor of adverse cardiovascular events in patients with DN [29]. eGFR is considered to be an important marker of renal status in DN [30]. The results of this study revealed that UAE and eGFR levels were consistent with those reported in previous studies. In addition, our results confirmed that miR-638 was significantly correlated with the progression of DN. Moreover, inflammation is considered to be a key factor in the pathogenesis of DN [31]. IL-1 is associated with increased permeability of vascular endothelial cells. IL-6 promotes tubule-interstitial neutrophils infiltration, which is associated with the thickening of the glomerular basement membrane. TNF-α is toxic to kidney cells and increases oxidative stress [32]. We found that IL-1β, IL-6, and TNF-α were overexpressed in the serum of patients with DN, which is consistent with the results of previous studies. In addition, miR-638 has abnormal regulation in inflammation-related diseases. The expression of miR-638 is significantly reduced in chronic inflammatory Behçet’s disease [33]. miR-638 is abnormally expressed in the kidneys of patients with lupus nephritis and is associated with the severity of clinical disease [16]. Our results confirmed a negative correlation between miR-638 and pro-inflammatory factors, suggesting that low expression of miR-638 is involved in the progression of DN through the regulation of inflammation.

Hyperproliferation and hypertrophy of HMC are some of the early symptoms of patients with DN [34]. and HG stimulation of HMC has been widely used in the study of DN in vitro [35]. Therefore, we exposed the cultured HMCs under HG to simulate hyperglycaemia to further evaluate the effect of miR-638 on proliferation and inflammation. The results confirmed that HG significantly inhibited the expression of miR-638, and induced the proliferation of HMCs. This was consistent with the results of previous studies, which reported that HG promoted the proliferation of HMC cells [36], whereas miR-638 mimic increased miR-638 levels and significantly alleviated HG-induced HMC proliferation. Furthermore, increased miR-638 levels were increased, it can significantly inhibit the increase in pro-inflammatory cytokines stimulated by HG. The results indicate that miR-638 may be involved in the progression of DN by regulating proliferation and inflammatory factors. In addition, inflammatory cytokine levels may be decreased owing to the decreased cell viability, thereby reducing the secretion of cellular inflammatory factors. This implies that miR-638 may reduce the expression of inflammatory factors by regulating cell proliferation and hence participate in the DN progression. However, in-depth research into the specific mechanism is required to verify this speculation, which will be the focus of our subsequent studies. Currently in clinical studies, although miRNA has not been used in DN treatment, the potential role of miRNA in the disease cannot be ignored. To ensure the safe application of miRNA therapy, some miRNAs have entered clinical trials [37].

Our results suggest that miR-638 is a potential miRNA candidate for the clinical treatment of DN, which may provide new insights into the diagnosis and treatment of DN. Identifying individuals with low miR-638 expression and early clinical intervention can prevent or delay the progression and complications of DN.

## Data Availability

Corresponding authors could provide data and materials if necessary.

## References

[1] Ogurtsova K, da Rocha Fernandes JD, Huang Y, Linnenkamp U, Guariguata L, Cho NH (2017). IDF Diabetes Atlas: Global estimates for the prevalence of diabetes for 2015 and 2040. Diabetes Res Clin Pract.

[2] Thomas MC, Cooper ME, Zimmet P (2016). Changing epidemiology of type 2 diabetes mellitus and associated chronic kidney disease. Nat Rev Nephrol.

[3] Raptis AE, Viberti G (2001). Pathogenesis of diabetic nephropathy. Exp Clin Endocrinol Diabetes.

[4] Zhang XX, Kong J, Yun K (2020). Prevalence of diabetic nephropathy among patients with type 2 diabetes mellitus in china: a meta-analysis of observational studies. J Diabetes Res.

[5] Martinez B, Peplow PV (2019). MicroRNAs as biomarkers of diabetic retinopathy and disease progression. Neural Regen Res.

[6] Yamagishi S, Fukami K, Ueda S, Okuda S (2007). Molecular mechanisms of diabetic nephropathy and its therapeutic intervention. Curr Drug Targets.

[7] Navarro-Gonzalez JF, Mora-Fernandez C, Muros de Fuentes M, Garcia-Perez J (2011). Inflammatory molecules and pathways in the pathogenesis of diabetic nephropathy. Nat Rev Nephrol.

[8] Loeffler I, Wolf G (2015). Epithelial-to-mesenchymal transition in diabetic nephropathy: fact or fiction?. Cells.

[9] Fineberg D, Jandeleit-Dahm KA, Cooper ME (2013). Diabetic nephropathy: diagnosis and treatment. Nat Rev Endocrinol.

[10] Conserva F, Barozzino M, Pesce F, Divella C, Oranger A, Papale M (2019). Urinary miRNA-27b-3p and miRNA-1228-3p correlate with the progression of Kidney Fibrosis in Diabetic Nephropathy. Sci Rep.

[11] Sedding DG, Boyle EC, Demandt JAF, Sluimer JC, Dutzmann J, Haverich A (2018). Vasa vasorum angiogenesis: key player in the initiation and progression of atherosclerosis and potential target for the treatment of cardiovascular disease. Front Immunol.

[12] Zhang Y, Sun Y, Peng R, Liu H, He W, Zhang L (2018). The long noncoding RNA 150Rik promotes mesangial cell proliferation via miR-451/IGF1R/p38 MAPK signaling in diabetic nephropathy. Cell Physiol Biochem.

[13] Campion CG, Sanchez-Ferras O, Batchu SN (2017). Potential role of serum and urinary biomarkers in diagnosis and prognosis of diabetic nephropathy. Can J Kidney Health Dis.

[14] El-Samahy MH, Adly AA, Elhenawy YI, Ismail EA, Pessar SA, Mowafy ME (2018). Urinary miRNA-377 and miRNA-216a as biomarkers of nephropathy and subclinical atherosclerotic risk in pediatric patients with type 1 diabetes. J Diabetes Complications.

[15] Delic D, Eisele C, Schmid R, Baum P, Wiech F, Gerl M (2016). Urinary Exosomal miRNA Signature in Type II Diabetic Nephropathy Patients. PLoS ONE.

[16] Lu J, Kwan BC, Lai FM, Tam LS, Li EK, Chow KM (2012). Glomerular and tubulointerstitial miR-638, miR-198 and miR-146a expression in lupus nephritis. Nephrology (Carlton).

[17] Luque A, Farwati A, Krupinski J, Aran JM (2018). Association between low levels of serum miR-638 and atherosclerotic plaque vulnerability in patients with high-grade carotid stenosis. J Neurosurg.

[18] American DA (2016). Erratum Classification and diagnosis of diabetes. Sec. 2. In Standards of Medical Care in Diabetes-2016. Diabetes Care 2016;39(Suppl 1):S13-S22. Diabetes Care.

[19] Gheith O, Farouk N, Nampoory N, Halim MA, Al-Otaibi T (2016). Diabetic kidney disease: world wide difference of prevalence and risk factors. J Nephropharmacol.

[20] Koya D, Haneda M, Inomata S, Suzuki Y, Suzuki D, Makino H (2009). Long-term effect of modification of dietary protein intake on the progression of diabetic nephropathy: a randomised controlled trial. Diabetologia.

[21] Wang LP, Gao YZ, Song B, Yu G, Chen H, Zhang ZW (2019). MicroRNAs in the progress of diabetic nephropathy: a systematic review and meta-analysis. Evid Based Complement Alternat Med.

[22] Mafi A, Aghadavod E, Mirhosseini N, Mobini M, Asemi Z (2018). The effects of expression of different microRNAs on insulin secretion and diabetic nephropathy progression. J Cell Physiol.

[23] Tang J, Yao D, Yan H, Chen X, Wang L, Zhan H (2019). The Role of MicroRNAs in the pathogenesis of diabetic nephropathy. Int J Endocrinol.

[24] Wang J, Duan L, Tian L, Liu J, Wang S, Gao Y (2016). Serum miR-21 may be a potential diagnostic biomarker for diabetic nephropathy. Exp Clin Endocrinol Diabetes.

[25] Abdelsalam M, Wahab AM, El Sayed ZM, Motawea M (2020). MicroRNA-451 as an early predictor of chronic kidney disease in diabetic nephropathy. Int J Nephrol.

[26] Zhao P, Zhang BL, Liu K, Qin B, Li ZH (2018). Overexpression of miR-638 attenuated the effects of hypoxia/reoxygenation treatment on cell viability, cell apoptosis and autophagy by targeting ATG5 in the human cardiomyocytes. Eur Rev Med Pharmacol Sci.

[27] Jiao W, Zhang D, Wang D, Xu R, Tang L, Zhao M (2019). MicroRNA-638 inhibits human aortic valve interstitial cell calcification by targeting Sp7. J Cell Mol Med.

[28] Haimoto H, Sasakabe T, Umegaki H, Wakai K (2012). Reduction in urinary albumin excretion with a moderate low-carbohydrate diet in patients with type 2 diabetes: a 12-month intervention. Diabetes Metab Syndr Obes.

[29] Ito H, Komatsu Y, Mifune M, Antoku S, Ishida H, Takeuchi Y (2010). The estimated GFR, but not the stage of diabetic nephropathy graded by the urinary albumin excretion, is associated with the carotid intima-media thickness in patients with type 2 diabetes mellitus: a cross-sectional study. Cardiovasc Diabetol.

[30] Tofte N, Suvitaival T, Ahonen L, Winther SA, Theilade S, Frimodt-Moller M (2019). Lipidomic analysis reveals sphingomyelin and phosphatidylcholine species associated with renal impairment and all-cause mortality in type 1 diabetes. Sci Rep.

[31] Zhao Y, Chen SJ, Wang JC, Niu HX, Jia QQ, Chen XW (2015). Sesquiterpene lactones inhibit advanced oxidation protein product-induced MCP-1 expression in podocytes via an IKK/NF-kappaB-dependent mechanism. Oxid Med Cell Longev.

[32] Perez-Morales RE, Del Pino MD, Valdivielso JM, Ortiz A, Mora-Fernandez C, Navarro-Gonzalez JF (2019). Inflammation in diabetic kidney disease. Nephron.

[33] Alipour S, Nouri M, Sakhinia E, Samadi N, Roshanravan N, Ghavami A (2017). Epigenetic alterations in chronic disease focusing on Behcet's disease: Review. Biomed Pharmacother.

[34] Ortega A, Romero M, Izquierdo A, Troyano N, Arce Y, Ardura JA (2012). Parathyroid hormone-related protein is a hypertrophy factor for human mesangial cells: Implications for diabetic nephropathy. J Cell Physiol.

[35] Wang Y, Zhao M, Zhang Y, Li X, Wang H (2002). Serum IgA(1) from patients with IgA nephropathy induces phosphorylation of extracellular signal-regulated kinase and proliferation of human mesangial cells. Zhonghua Yi Xue Za Zhi.

[36] Cao Y, Cao X, Sun L, Li Y (2019). miR-206 Inhibits Cell Proliferation and Extracellular Matrix Accumulation by Targeting Hypoxia-Inducible Factor 1-alpha (HIF-1alpha) in Mesangial Cells Treated with High Glucose. Med Sci Monit.

[37] Linna-Kuosmanen S, Tomas Bosch V, Moreau PR, Bouvy-Liivrand M, Niskanen H, Kansanen E (2021). NRF2 is a key regulator of endothelial microRNA expression under proatherogenic stimuli. Cardiovasc Res.

